# “It’s very saddening, you keep on wondering when the symptoms will be over”: A qualitative study exploring the long-term chikungunya disease impact on daily life and well-being, 6 years after disease onset

**DOI:** 10.1371/journal.pntd.0011793

**Published:** 2023-12-06

**Authors:** Churnalisa Doran, Ashley Duits, Adriana Tami, Izzy Gerstenbluth, Ajay Bailey

**Affiliations:** 1 University of Groningen, University Medical Center Groningen, Department of Medical Microbiology and Infection Prevention, Groningen, The Netherlands; 2 Curaçao Biomedical and Health Research Institute, Willemstad, Curaçao; 3 Red Cross Blood Bank Foundation, Willemstad, Curaçao; 4 Department of Immunology, Curaçao Biomedical and Health Research Institute, Willemstad, Curaçao; 5 Institute for Medical Education, University Medical Center Groningen, Groningen, The Netherlands; 6 Department of Epidemiology, Curaçao Biomedical and Health Research Institute, Willemstad, Curaçao; 7 Department of Human Geography and Spatial Planning, University of Utrecht, Utrecht, The Netherlands; Tufts Medical Center, UNITED STATES

## Abstract

**Background:**

Long-term chikungunya is a mosquito-borne disease, characterized by disabling rheumatic symptoms persisting for years, after infection with the chikungunya virus. Previous studies focused on assessing the well-being of affected individuals from a quantitative perspective using generic instruments, and have reported physical and psychological impairment. However, a common critique is that generic instrument’s structured responses and pre-defined health domains selected by health professionals, may not capture the full extent of well-being impairment experienced by patients. This study aimed to explore in-depth to which extent long-term chikungunya disease impacts daily living and the physical, psychological, and social well-being from the experiences and perspective of affected individuals.

**Methodology/Principal findings:**

Using open-ended questions, in-depth interviews were conducted with 20 purposively selected individuals with long-term chikungunya disease, in Curaçao. Interview audio-recordings were transcribed verbatim. The data were thematically analyzed. Living with persistent rheumatic symptoms affected the participant’s daily living and well-being in several ways: experience of physical impact (restricted physical functioning and limitations in activities of daily life); experience of psychological impact (altered emotional state, fear of walking and running, psychosocial aspects of footwear adaptations, and uncertainty about disease progression and future health); and experience of social impact (social isolation and impaired relational maintenance, social dependency, challenges of social support, at-work productivity loss, and giving up leisure activities after work).

**Conclusions/Significance:**

This study, the first of its kind, indicated that the adverse impact of long-term chikungunya disease is currently underreported. The persistent rheumatic symptoms had a negative effect on functional ability, which in turn impacted broad aspects of daily life and well-being, beyond what is captured by generic instruments. In the view of the findings, physical exercise programs including manual therapy, aerobics, resistance and stretching exercises, and orthopaedic footwear interventions in a multidisciplinary patient-centred approach may improve physical function and subsequently overall well-being.

## Author summary

Long-term chikungunya disease is characterized by persistent rheumatic symptoms for years, after infection with the mosquito-borne chikungunya virus. Quantitative studies using generic instruments, consisting of pre-selected structured responses, reports physical and psychological well-being impairment among affected individuals. Qualitative studies using open-ended questions exploring long-term chikungunya disease impact on daily living and well-being from the experiences of affected individuals are scarce. This qualitative study aimed to fill this gap. In our study, physical functioning restrictions induced by rheumatic symptoms impaired well-being beyond what has been captured by generic instruments. These restrictions led to inactivity, struggle to avoid weight gain, and performance of domestic tasks in stages. Next to an altered emotional state, a high level of fear to walk or run was experienced, which required unwilling footwear adaptations for comfort and support, causing subsequent emotional impairment. The restricted physical functioning also reduced social participation and interest in social relations, but also introduced dependency on others. In addition, presenteeism and decreased productivity at work, and giving up leisure activities after work to rest were reported. To improve physical functioning and subsequent overall well-being, multi-disciplinary physical exercise programs including manual therapy and stretching exercises, and orthopaedic footwear interventions could be implemented.

## Introduction

Long-term chikungunya disease is a mosquito-borne disease caused by the re-emerging chikungunya virus (CHIKV), which has infected more than 10 million individuals worldwide in the last two decades [1]. The disease mimics rheumatoid arthritis (RA) and is characterized by constant or recurrent rheumatic symptoms of moderate to severe pain including severe poly-arthralgia, joint swelling and stiffness, and myalgia prominently in the extremities, which can persist for years [2,3]. Due to the scarce etiological knowledge, there is currently no curative treatment or agreement regarding appropriate therapy for disease management [4,5], seriously affecting the health-related quality of life (QoL), which can be defined as impact of a disease on the ability of a person to function and perceived well-being in physical, psychological, and social aspects of life [6]. The perceived well-being is recognized as increasingly significant for patients affected by long-term diseases or chronic pain as physical function decreases [7]. A significant amount of quantitative studies using the SF-36 or SF-12 generic QoL instruments, have reported long-term chikungunya disease impairment in the physical and psychological domains [8–11]. Although, frequently used in research, especially in comparative studies, generic instruments have been questioned for to what extent structured responses and pre-defined health domains, selected by health professionals actually captures or represent the well-being of individual patient’s [12]. Relatively little emphasis has been placed on defining the long-term chikungunya disease impact on daily life and well-being from direct in-depth exploration of the individual’s personal experiences. As far as we are aware of, only one qualitative study evaluated the well-being of affected individuals, one year after CHIKV infection [13]. Our qualitative study aimed to fill this gap, by assessing to which extent rheumatic symptoms induced by long-term chikungunya disease impact the daily living and perceived well-being in physical, psychological, and social aspects of life among affected individuals using an experience based approach. As there is yet no specific treatment, this may guide disease management interventions and allocate health-care resources closely matched to the difficulties that are directly experienced, to diminish the disease burden and improve the overall well-being of those affected.

## Methods

### Ethics statement

The study was approved by the Medical Ethical Committee of the Saint Elisabeth Hospital in Curaçao (Reference number: 2015–002). Participants received oral and written information about the study and signed a written informed consent. Confidentiality and anonymity were ensured by assigning a code to each participant.

### Study site and population

Chikungunya virus emerged in the Americas in December 2013, on the Caribbean island of Saint-Martin [14]. Since the first autochthonous transmission in Saint-Martin, CHIKV has been identified in 45 countries and regions in the Americas, including Curaçao, causing an estimated >3 million confirmed and suspected cases [15]. Curaçao is an autonomous island state within the Kingdom of the Netherlands, located in the Caribbean [16]. In June-July 2014, CHIKV emerged in Curaçao for the first time, infecting approximately 30–50% of the 150.000 inhabitants. The epidemiology of CHIKV in Curaçao has been described in our previous studies [11,17]. In 2015, a prospective longitudinal chikungunya cohort study was established, to follow the long-term clinical manifestation and burden of CHIKV in patients. Chikungunya infection was diagnosed on the presence of acute febrile symptoms and/or positive CHIKV laboratory test [18].

### Study design and participant

The consolidated criteria for reporting qualitative research (COREQ) were followed [19]. This qualitative exploratory study comprised of 20 adults, was embedded in the longitudinal chikungunya cohort study. Qualitative methodologies are explicitly developed to investigate experiences from the perspectives of individuals using open-ended questions [20]. In August 2020, using cohort records extracted from a follow-up health survey conducted between July 2019 and March 2020 [11], 51 female and 9 male patients who self-declared being affected by persistent rheumatic symptoms and having no joint problems and/or mental health conditions prior to CHIKV infection were identified and considered potentially eligible. The gender imbalance corresponds with epidemiological data, showing females to be more affected by long-term chikungunya disease compared to males [21]. A maximum variation purposive sampling technique was employed based on gender, age, and a range of persistent rheumatic symptoms for participant selection, to allow for a broad range of long-term chikungunya disease impact on daily living and well-being [22]. In September and October 2020, the selected patients were contacted by telephone and invited to participate on the basis that they met the inclusion criteria of still experiencing persistent rheumatic symptoms, at the time of contact. Out of the 9 male patients approached, 6 self-declared being recovered from persistent rheumatic symptoms and were excluded. The inclusion of participants continued until data saturation was achieved [23].

### Data collection

In September and October 2020, the first author CD (Master in Biomedical Sciences/ PhD candidate), a female researcher with experience conducting interviews and fluent in the native local language Papiamentu, conducted all in-depth interviews to guarantee both credibility and homogeneity. The first author had professional contact as an interviewer with 3 of the included participants during quantitative data collection of the cohort [11]. The in-depth interviews were conducted at home or work according to participant’s preference with no other person attending. A piloted semi-structured topic guide was developed based on literature review that identified important open-ended questions on physical, psychological, and social well-being, which were discussed during the planning phase of the study with AB (professor in Social Urban Transitions), an anthropologist and senior qualitative researcher. The topic guide consisted of five main areas: (i) persistent rheumatic symptoms since acute symptom onset and their locations; (ii) influence of persistent symptoms on physical and psychological well-being; (iii) interference of persistent symptoms with daily functioning and social life; and (iv) health expectations (see S1 File). Using follow-up questions and probes the real-life experiences and perceived well-being were explored. In addition, paraphrasing and summarizing main points was employed to clarify ambiguous statements and minimize misunderstandings. Participants were also given the opportunity to discuss new topics that they considered relevant. Interviews lasted between 25–75 minutes and were audio-recorded. Data saturation was reached after 16 interviews, with the final additional 4 interviews being conducted for confirmation [23]. All audio-recordings were transcribed verbatim by a paid independent transcriber. No repeat interviews were carried out and transcripts were not returned to participants.

### Data analyses

The qualitative software program Atlas.ti was used for data management, which were analysed using an inductive thematic analysis approach [24], in Papiamentu to preserve linguistic meanings. The transcripts were revised for accuracy and re-read for familiarization by CD. Using line-by-line open codification, statements related to daily life experiences and well-being were coded in English and categorized into physical, psychological, and social well-being [24]. Initial codes were refined following continuous comparison of the transcripts. For quality assurance, AB independently checked two random transcripts, which were translated to English. Subsequently, CD and AB revised and discussed the initial codes, before the codes were collated into potential themes by CD. The themes were reviewed and analysed by repeated revision of the transcripts, until themes saturation [23], after which CD and AB met again for consensus on the final themes. Relevant quotes were translated to English by CD to add further depth to the themes, and were altered grammatically to improve readability, where necessary [24].

## Results

All invited patients consented to participate, 17 females and 3 males, aged 32 to 77 years, with disease duration ranging between 68 and 74 months (6 years). The most frequently reported persistent rheumatic symptom was (poly)arthralgia followed by joint weakness, stiffness, swelling, cramps and locking, and myalgia, which were experienced daily, every other day or on a weekly basis. The socio-demographic and rheumatic characteristics of the participants are shown in Table 1.

**Table 1:** Socio-demographic and rheumatic characteristics of participants.

ID	Age	Gender	Employment	Relationship status	Co-habitants (n)	Disease duration (months)	Rheumatic symptoms and locations
Pt. 1	56	Female	Employed	Married	4	71	Arthralgia in ankles
Pt. 2	45	Female	Employed	Unknown	Unknown	73	Arthralgia in shoulder, wrists and ankles, and joint weakness in wrists and ankles
Pt. 3	55	Female	Employed	Single	3	70	Arthralgia in knees and joint swelling in knees and ankles
Pt. 4	57	Male	Employed	In a relationship	5	69	Joint cramps in hands and feet
Pt. 5	50	Female	Employed	In a relationship	2	70	Joint stiffness in ankles
Pt. 6	66	Female	Retired	Married	8	73	Arthralgia and joint stiffness in knees
Pt. 7	54	Female	Employed	In a relationship	4	70	Arthralgia in hands, fingers, hips, knees, and ankles
Pt. 8	60	Male	Employed	Married	2	73	Arthralgia, joint swelling and stiffness in the fingers
Pt. 9	44	Female	Employed	In a relationship	2	71	Arthralgia in the back and fingers, and joint weakness in wrists and hands
Pt. 10	77	Female	Retired	Single	1	70	Arthralgia in shoulder, knee and toe, joint stiffness in knee, and joint swelling in ankle
Pt. 11	35	Female	Unemployed	Single	3	74	Arthralgia in the wrists, hands, and knee
Pt. 12	48	Female	Employed	In a relationship	4	70	Arthralgia and joint weakness in wrists and hands, and joint stiffness in knees
Pt. 13	56	Female	Employed	Unknown	5	72	Arthralgia and joint stiffness in the lower back and ankles
Pt. 14	65	Female	Retired	Single	2	71	Joint weakness in wrist and finger
Pt. 15	48	Female	Unemployed	Divorced	3	72	Arthralgia in elbow, wrist and knee, and joint weakness in wrist and knee
Pt. 16	32	Female	Employed	Married	2	72	Arthralgia and joint stiffness in the lower back and knee
Pt. 17	56	Female	Employed	Single	1	72	Arthralgia in wrists, fingers, knees and ankles, joint locking of wrists, fingers and ankles, joint cramps in fingers, and joint swelling of ankles
Pt. 18	58	Male	Employed	In a relationship	2	73	Myalgia in thigh and hamstring
Pt. 19	49	Female	Employed	In a relationship	2	70	Arthralgia and joint weakness in wrists and ankles
Pt. 20	62	Female	Employed	Single	1	73	Arthralgia and joint cramps in wrist, and joint stiffness in hips, knees, and ankles

Living with long-term chikungunya disease affected the participants’ well-being in several ways. In the following, the reported daily life experiences and perceived impact of disease in their physical, psychological, and social well-being, which are divided into multiple themes will be described. The code lists (themes, codes, and illustrative quotes) related to the experience of physical, psychological, and social impact are shown in S1, S2 and S3 Tables, respectively.

### 1. Experience of physical impact

The experiences of the participants indicated impairment or restrictions in everyday physical ability and their ability to perform activities of daily living. Experience of physical impact described the negative effect of persistent rheumatic symptoms on physical functioning and activities of daily life (ADL).

#### 1.1. Restricted physical functioning

Participants described a range of physical function restrictions in the upper extremities like arm and hand mobility, including reaching, lifting and carrying objects, twisting the wrist, or fine motor skills, such as grip force or squeezing. One participant with limited grip force due to arthralgia, and joint swelling and stiffness in the fingers explained:

*“When a [bottle] cap is for example small, you’ll have more trouble [to open the bottle], because you cannot really make a strong grip. If the cap is a little bigger, then you can [make a strong grip], when its smaller, it’s harder. […]. When you need to open a small cap, it’s difficult, because you need to really force, because you cannot close your hand to put strength [on the cap].”*

*(Pt. 8)*

Restrictions related to the lower extremities included moving and walking, such as sitting up or down or bending positions, walking up or down the stairs, walking moderate or fast, and walking long distances. Another participant expressed how joint stiffness after inactivity impairs walking ability:

*“You know especially when I sit down and I need to stand up, I feel that it [ankles] is stiff to walk on. I can walk normal, but when I sit down for too long and I stand up, I will feel that it [ankles] is stiff in the joints for it [ankles] to move. […]. It also hampers me when I walk, so conditional walk [moderate or fast pace].”*

*(Pt. 5)*

#### 1.2. Limitation in activities of daily life

The rheumatic symptoms and physical restrictions made it difficult to participate in ADL. Some participants complained about the lack of restful sleep and almost all experienced morning stiffness. In relation to personal care, some participants encountered challenges in getting dressed and eating. Participants were also limited in managing or maintaining physical fitness, which led to an ongoing battle with weight gain, as one participant with arthralgia and joint weakness in the ankles stated:

*“I used to walk [conditional walk] but I don’t go [for walks] anymore, that’s what made me gain weight. I am not a person that will go on a diet, I used to eat and then go for a walk, and stay in shape [managing physical fitness]… but now I don’t go for a walk anymore and I keep gaining weight.”*

*(Pt. 19)*

Some participants expressed investing in appliances such as a lightweight iron, mopping system with wringer, or washing machine with spinner for pain relieve and to ease everyday life. In addition, normal domestic tasks such as cleaning, doing the laundry, gardening, taking care of pets, and cooking needs extra effort, more time to perform, or were performed in stages. One participant expressed how her physical endurance in performing domestic tasks has become impaired, as experienced by many participants:

*“I was very active [pre-infection], you [interviewer] can see how many birds [in cages] and plants I have [in the garden]…but now I cannot do everything [domestic tasks] at once [now performed in stages], I am much slower. I used to have a lot of speed, not a thing [domestic task] lasted long to do, but now I need to take more time.”*

*(Pt. 9)*

### 2. Experience of psychological impact

The experiences of living with persistent rheumatic symptoms were perceived as having a negative psychological impact, which involved a general state of distress, described by four relevant themes: altered emotional state, fear of walking and running, psychosocial aspects of footwear adaptations, and uncertainty about disease progression and future health.

#### 2.1. Altered emotional state

Facing ongoing pain and loss of physical ability, shaped the participant’s emotional well-being. For some, the persistent symptoms had profoundly altered their emotional state. They described becoming irritable, stressed, moody, bad tempered, or unhappy. One participant explained how persistent arthralgia changes her emotional state:

*“It [joint pain] will not go away, it keeps bothering me, bothering me, I feel that my mood changes, I don’t want to be bad tempered.”*

*(Pt. 17)*

The recurrent nature and perceived triggers of rheumatic symptoms also altered the emotional state. Some participants described feeling frustrated when symptoms re-occurred or fear and anxiety when getting the flu, which was perceived as triggering symptoms or increased pain intensity. One participant described how the arthralgia in her knee intensifies when getting the flu:

*“I always get the flu with body ache. […]. The pain is very severe and it affects me more in my knee [joint affected by chikungunya disease]…and when I am standing while I have the flu, the pain in my knee will concentrate [increase]. […]. When I feel that I will get the flu, I will immediately drink vitamins! […]. I am really afraid [of getting the flu].”*

*(Pt. 12)*

#### 2.2. Fear of walking and running

Moreover, due to perceived joint instability and weakness in the knees and ankles participants were very vigilant, fearful and anxious to not trip or fall when walking. One participant with arthralgia and joint weakness in the ankles expressed:

*“In the ankle, in the ankle I’ve noticed there is instability [joint weakness], I need to watch out very well [when walking]. Very easily the instability can make me twist my ankle, so I need to watch out very well. That [instability] is what I noticed that is happening with regularity.”*

*(Pt. 2)*

Another participant mentioned experiencing fear on a daily basis, expressing that if something life-threatening happens, you aren’t able to run to save your life, because of the rheumatic symptoms:

*“In the morning, I am like [swear word]. […]. If something that requires to run happens, I cannot run. I feel as if the two things [ankle and foot] in my lower leg aren’t connected [instable], therefore I cannot run, so every day I think that if something that requires running happens, what will I do? […]. If I need to run to save my life, I cannot run.”*

*(Pt. 19)*

#### 2.3. Psychosocial aspects of footwear adaptations

For many female participants persistent symptoms in the lower extremities induced restrictions in footwear selection and required unwilling footwear adaptations including the shoe type or size, height or width, and sole types for comfort, balance, and support. These restrictions impacted their emotions negatively. One female participant expressed the feeling of hopelessness in her attempts to find comfortable footwear:

*“If I wear shoes that aren’t orthopaedic, I will get affected [pain] in my feet. Lately, I have noticed that even the orthopaedic shoes that I pay a lot of money for will make my feet hurt…so eventually I don’t know what else I can do…so I can wear a shoe that does not bother [hurt] me.”*

*(Pt. 1)*

The shoe choice of these participants was now based on their current health status rather than what they would desire or choose to wear. The adaptations and loss of choice in footwear was perceived as a daily struggle or restriction, especially when social events needed to be attended. One participant with joint swelling in the ankles expressed:

*“With regards to my social and emotional well-being, it [loss of footwear choice] is really disheartening. When I go out [attending social events], I need to watch out very carefully [taking symptoms into account], my shoes need to be a block heel or streamline [for stability] and for sure two sizes bigger [for the shoe to fit]. I used to wear eight [shoe size] and now I wear ten [shoe size]. […]. I loved to wear high heels [stiletto heels] when going out.”*

*(Pt. 3)*

#### 2.4. Uncertainty about disease progression and future health

Participants expressed being uncertain, questioning if they will return to pre-chikungunya health status, and worried about the disease progression in combination with disabilities associated with increasing age.

*“I don’t think that my situation [having persistent symptoms] will improve. […]. I will deal with them [persistent symptoms], but I don’t think they will improve at this age. I don’t think I will be pain free…without anything [complains].”*

*(Pt. 10)*

Others were hopeful and optimistic, hoping that their symptoms will subside or won’t get worse in pain intensity. The remission of symptoms was also perceived as an indication of disease improvement. However, on participant stated that symptom remission can be deceitful and give false hope of entering the recovery trajectory:

*“I cannot remember if it was an interview [cohort follow-up survey] in 2017 or 2018 that I responded that I was not affected that much, but fast forward, after 3 years, the pain in my ankles is the one that I’m currently most affected by. It went away, I did not feel anything. I thought…hey [impression when something is realized] I’ve recovered…but it came back.”*

*(Pt. 19)*

### 3. Experience of social impact

The majority of the participants mentioned having an active social life prior to CHIKV infection, but many experienced the debilitating physical symptoms and/or emotional distress as having a socially limiting effect on their lives. The themes identified were: social isolation and impaired relational maintenance, social dependency, challenges of social support, at-work productivity loss, and giving up leisure activities after work.

#### 3.1. Social isolation and impaired relational maintenance

Due to loss of physical functioning or pain, some participants were not able to participate in activities such as group hiking, dancing, or having meals out with friends, and preferred staying at home and isolated themselves socially.

*“I don’t want to deal with anybody, I don’t want to see anybody, I don’t want to go out [participating in social activities]. […]. Because truly, believe me, when you [talking in third person] have that pain you don’t want to see anybody, you don’t want to do this or that [social activities]…the telephone is ringing, you don’t want to pick up, you will check first who is calling before you pick up. It isn’t nice, it isn’t nice at all.”*

*(Pt. 17)*

Some participant described losing interest to maintain social relationships due to pain and discomfort. Sometimes to the extreme that they disregarded visitors or having a tendency the vent their frustration on others.

*“Of course it [persistent symptoms] has an effect on you, because you want to be well…you don’t want to feel this or that [having persistent symptoms]. It makes me nervous, it makes me asocial. I prefer to get rid [distancing] of you [social relationship], because I’ll treat you bad.”*

*(Pt. 20)*

#### 3.2. Social dependency

Social support identified involved dependency on family and friends, including receiving help with domestic tasks and bringing meals, when participants experienced symptoms. Asking for practical help also enabled participants to avoid performing tasks which caused or worsened pain. One participant with arthralgia, joint locking and cramps in the wrists and fingers explained:

*“I have a great-aunt that I take care of, so I have to do groceries for her when I do mine. I need to ask a girlfriend to go with me, because pushing a [heavy] shopping trolley will be a problem [painful] for me.”*

*(Pt. 17)*

Moreover, one participant with arthralgia, joint stiffness and swelling in the knee and ankle described being dependent on companionship to feel safe and not to fall victim to crime during social activities:

*“I used to go to the movies alone, but I don’t go alone anymore…now I will go with a girlfriend. That isn’t only because of chikungunya, the times became bad, and for me that has this [persistent symptoms] and walks slowly slowly, people may see me and say…that person is walking bad, this and that, let’s rob her. That’s why I don’t go alone anymore.”*

*(Pt. 10)*

#### 3.3. Challenges of social support

The occurrence of negative influences in social relationships was also reported, including unsupportive behaviours and attitudes. Participants expressed that individuals who did not have chikungunya cannot understand or fully comprehend how persistent symptoms still impacts their daily lives. Some experienced being met with sceptical and distrustful responses. One participant with arthralgia and joint stiffness in the lower back and knee expressed her frustration when dealing with sceptical attitudes:

*“I have these symptoms since I got that thing [chikungunya]. People who did not get it [chikungunya] will not understand. […]. When I bend down to get something, I will say…ayyy [expressing being in pain], they [others] will say…you are a young child [being sceptical of painful joints because of young age]. I will answer that I know that I am young, but chikungunya does not care about my age!”*

*(Pt. 16)*

Participants also mentioned being ridiculed or labelled as an exaggerator of symptom intensity and having their symptoms downplayed by their social network, especially due to lack of general awareness and knowledge of possible persistency of severe symptoms after CHIKV infection. One participant expressed:

*“I have a colleague that would laugh when I stood up from a chair after chikungunya. After I got it [chikungunya] for a while, she got it…She told me…Hombuuuu [expression being very surprised or overwhelmed] he eh [expression finding something unfavourable or bad]. I told her…Yes when I stood up you were laughing, maybe now you feel [the pain] what I am feeling. Till this day they [colleagues] will laugh when I stand up [being ridiculed], it’s a joke [for colleagues], it’s really a joke.”*

*(Pt. 9)*

#### 3.4. At-work productivity loss

The majority of the participants were employed at the time of interview and did not miss work days. However, persistent symptoms appeared to play a major role in occupational function. Participants working in both manual and office environments reported presenteeism, for not being able to function optimally or that work related duties aggravated symptoms. One participant with arthralgia in the wrists and hands explained:

*“I work as a waiter [in a restaurant] and it [symptom] has become a problem, but because I am a person that does not give up, I continued working with my hand in that way [hand folded backwards with hand palm up]…even if it [holding tray] causes a lot of pain in my wrist.”*

*(Pt. 11)*

Participants described difficulties in standing or being sedentary for a certain amount of time, having the need to delegate their work to colleagues, slow down and take extra time, rest during work tasks, or take their time to stretch the joints, affecting their at-work productivity.

*“Normally, I would have continued continuously, but now I can not sit for a long period of time. […]. There are times that I feel a lot of pain in my bones [joints] and will decide to rest my body. If I am at work, I will stand up and walk back and forth or I will do the thing [work task] that I was doing less.”*

*(Pt. 9)*

#### 3.5. Giving up leisure activities after work

Work related descriptions also involved interference with social activities and dictated to which extent social activities after work was possible. For some participants, the persistent symptoms made it difficult to prioritize anything else than work and to give up leisure activities with family and others after work to rest. One participant with joint stiffness in hips, knees and ankles explained:

*“When I come home from work and if my body permits, I will go to my neighbours. If they [neighbours] come [visiting when the participant has joint stiffness after work] I will say no [to neighbours], not today.”*

*(Pt. 20)*

The same participant explained and demonstrated:

*“When I come home from work in the afternoon, I’m like a handicapped woman. I think that a 100 year old woman walks better than me. When I sit down and want to get up, I will hold on hold on [demonstrated holding to the chair to get up], take a shower, give the dog food, and go to bed…that’s my life now. […]. When you see me you will not believe that I was the lady full of life going to work in the morning…serious [not joking].”*

*(Pt. 20)*

## Discussion

The findings of this study add to the existing quantitative literature, by taking a qualitative and experience based approach in exploring long-term chikungunya disease impact on the daily life and well-being of affected individuals, 6 years after acute disease onset. (Poly)arthralgia, joint weakness, stiffness and swelling were the persistent rheumatic symptoms most commonly described as having a direct negative impact on physical functioning, this in turn impacted broad aspects of the physical, psychological, and social well-being profoundly. In addition, new findings of long-term chikungunya disease impact on well-being among affected individuals such as fear of walking and running, psychosocial aspects of footwear adaptations, weight gain avoidance, deliberate loosing interest to maintain social relationships, challenges of social support, and at-work productivity loss were identified.

The persistent rheumatic symptoms reported in our sample are similar to that found in previous chikungunya studies [25]. This study highlighted the heavy physical burden associated with persistent rheumatic symptoms on the functional capacities of the upper and lower extremities, in concordance with previous arthritis studies [26,27]. These physical restrictions led to inactivity, limited endurance to perform ADL, and an ongoing struggle to avoid weight gain. Previous chronic pain studies reported that inactivity and sedentary lifestyles predicted impaired physical and psychological functioning, increased pain related interference in ADL [28], and risk factors for body mass index increase [29]. Furthermore, our study reports for the first time how women affected by long-term chikungunya disease can be restricted in their footwear choice, due to poorly fitting or inadequate footwear that exacerbated complaints [30]. The limitation in the footwear choice impacted emotional well-being negatively, as has also been described by women with RA [31]. The impact on emotional well-being identifies footwear as a key personal attribute to the lives of these women and not just solely fashion accessories.

In addition, the longstanding suffering and physical restrictions altered emotional state and induced emotional distress [13,32], which are associated with increased pain intensity and the impact of illness on QoL [33]. Moreover, a high level of vigilance and/or fear to walk or run was experienced, due to the perceived instability and weakness of the joints in the lower extremities. Weakness of the lower extremity muscles due to disuse atrophy, has been observed in RA patients when normal physical functions are obstructed or certain pain aggravating movements are avoided [34,35], which may influence balance dysfunction.

Moreover, restriction in physical functioning or emotional distress reduced social participation and induced social isolation, consistent with other studies [13,32]. However, on the other hand, our study also confirms the importance of having social support for reorganizing household responsibilities, domestic tasks, and to resume pre-infection valued social activities [36–38]. In addition, negative social responses and behaviours, and lack of understanding were reported [39]. Rheumatic symptoms such as arthralgia, joint stiffness, cramps and weakness are inherently invisible. This invisibility, particularly accompanied by a lack of etiological knowledge of symptoms persistency after CHIKV infection, can provoke invalidation, which includes disbelief, discounting, lack of understanding, and negative responses from the social environment [40]. The perception that social support is unavailable during stressful events like a long-term disease has a direct negative effect on physical and psychological well-being [41]. Interestingly, our results describe the notion that some participants would deliberately not maintain social relationships, which is in contrast with previous chronic studies [42,43]. The aforementioned findings may indicate that not the social network size, but the quality of social support may be more valued [44,45]. Support groups run by peers in which disease related information and personal experiences are shared may have utility in helping affected individuals with adequate qualitative support to cope with long-term chikungunya disease and enhance QoL [46].

In addition, until now, little has been known about the work productivity loss of individuals with long-term chikungunya disease, 6 years after disease onset. Persistent symptoms were reported to have an impact on both presenteeism and work productivity, due to the tension between pain, limited physical capacity, work-related demands, and increased need of rest at work [47,48]. A decrease in work productivity has been associated with impaired psychological well-being [49]. It is therefore important to provide the opportunity to adjust the workload, hours, and tasks of individuals affected by long-term chikungunya disease according to their reduced physical capacity, in line with WHOs recommendations for health related workplace adjustments [50].

International disease management guidelines are not consensual regarding specific therapeutics or treatment strategy for persistent rheumatic symptoms following acute disease. In the Americas, the Brazilian Society of Rheumatology released recommendations for the therapeutic treatment of chikungunya fever with chronic joint manifestations, using the findings of studies conducted in Brazil, Martinique, Colombia and Dominican Republic. However, currently there is low quality of clinical trial evidence to support these recommendations [51]. Therefore, supportive disease management interventions including rehabilitation therapies according to the perception of disability felt by those affected should be investigated to establish more strategies to improve physical functionality and overall well-being.

Treatment recommendations can be formulated in the light of our findings. Physical functioning was expressed in this study as being crucial for maintaining mobility and being able to perform ADL, retain emotional well-being, social participation, as well as performing work related tasks. Properly designed physical exercise programs including manual therapy, aerobic, resistance and stretching exercises have shown to be fundamentally beneficial for RA patients [52–58], which included increased muscle mass, improved strength, physical functioning and foot health, reduced adiposity, and significant improvement of psychological well-being, without exacerbation of disease activity [56–58]. However, it is important to be aware of the significant role of impaired emotional well-being induced by persistent rheumatic symptoms, which may influence not only motivation but also compliance with treatment recommendations [59]. For example, anxious patients may fear engaging in what they perceive as harmful or demanding physical exercises. Thus, attending health-care professionals must focus on physical disabilities as well as on the emotional states of patients in a multi-disciplinary patient-centred treatment method. These individuals may require a more tailored treatment approach, such as graded exposure to physical exercises to disconfirm their fearful perceptions and beliefs [60]. In addition, orthopaedic footwear interventions may be a relevant treatment option to decrease discomfort and enhance QoL [31,61]. In future research, the perspectives on the importance and acceptability of physical exercise programs and orthopaedic footwear interventions should be explored.

The findings of this study should be viewed in light of some strengths and limitations. This study is the first that takes a qualitative and experience based approach in exploring long-term disease impact on daily living and physical, psychological, and social well-being, 6 years after disease onset. The explorative nature of the study supported in the understanding of the disease impact more broadly and in context. However, the findings may not be generalizable to a global population, as all participants were ethnically Afro-Caribbean and symptoms were self-declared and not verified by a clinician. Yet, the insights and experiences are likely to have transferable commonalities, as the range of reported persistent rheumatic symptoms is comparable to quantitative study reports of different ethnic groups, countries and regions across the globe [25]. In addition, the results from the study can be utilised for medical education about chikungunya disease sequalae but also for community information programs in affected countries and regions at risk for CHIKV transmission. The latter will empower patients or people living with long-term chikungunya to access better care options. Specifically for the Americas, the Pan American Health Organization recommends that Member Countries should make reporting autochthonous CHIKV transmission mandatory in surveillance systems to enable and promote timely response, considering the amount of travel between countries in the region [62]. However, these report systems available to all Member Countries can also be used for knowledge sharing and disease management to improve situational awareness and increase reaction capacity for designing and implement supportive disease management interventions collaboratively to improve the well-being of affected individuals. Further, 3 participants had previous professional contact with the interviewer, which could have influenced their responses. Finally, a single coder was used during data analysis, which may introduce the risk of bias. However, the sample size was adequate for a qualitative method, a piloted topic guide was used, and codes and themes were discussed with a senior qualitative researcher. These methodological aspects improve the credibility of the findings, which also triangulates with existing chronic pain and RA literature. Future research is needed to validate and build upon the preliminary findings outlined in this study. Specifically, mixed qualitative-quantitative research methods using generic instruments such as the Health Assessment Questionnaire, which assesses work disability should be employed.

In conclusion, the adverse impact of long-term chikungunya disease on well-being is currently underreported. This qualitative study presents an overview of physical, psychological, and social well-being impairment beyond what has been captured by quantitative studies using generic instruments, and provide health professionals with evidence that can inform disease management interventions. Physical exercises such as manual therapy and stretching exercises and orthopaedic footwear interventions in the treatment of long-term chikungunya disease in a multi-disciplinary patient-centred care approach may improve physical function and subsequently overall well-being.

## Supporting information

S1 File Interview topic guide. (DOCX)

S1 Table Code list: Themes, codes, and illustrative quotes related to experiences of physical impact. (DOCX)

S2 Table Code list: Themes, codes, and illustrative quotes related to experiences of psychological impact. (DOCX)

S3 Table Code list: Themes, codes, and illustrative quotes related to experiences of social impact. (DOCX)
