# Supplementary material for: “It’s very saddening, you keep on wondering when the symptoms will be over”: A qualitative study exploring the long-term chikungunya disease impact on daily life and well-being, 6 years after disease onset
Source: PLoS Negl Trop Dis. 2023 Dec 6;17(12):e0011793. doi: 10.1371/journal.pntd.0011793 (PMC10699624; doi:10.1371/journal.pntd.0011793)
Supplement: S1 File — (DOCX) [file pntd.0011793.s001.docx]

**S1 File. Interview topic guide.**

1. Could you introduce yourself, what is your age, the amount of people living with you in your household, and occupation?

2. Would you please describe your experience with chikungunya disease, starting from the first symptoms?

3. What are the symptoms that you are still experiencing, since chikungunya infection?

1. Describe the rheumatic symptoms; joint pain, swelling, stiffness, cramps and/or locking, and body locations.
2. Are the rheumatic symptoms constant or recurrent (come and go)?
3. How long will the recurrent symptoms last?

4. What effect do the chikungunya related symptoms have on you? Can you explain why?

1. Describe the personal effects; physically and emotionally (thoughts and feelings).
2. Describe the effects on family, social-network, and work; relationships, recreational activities, and occupational duties.

5. In which way does the chikungunya related symptoms and pain interferes with your daily functioning and activities? Can you explain how?

6. In which way does your social network (family, friends, or colleagues) understand that you have persistent symptoms?

1. Have you asked or received support?

7. How healthy do you feel living with chikungunya disease? Can you explain why?

1. Is there a difference between before chikungunya and now years after infection?
2. How is your physical and emotional health?

8. How do you see your future health with regards to living with chikungunya disease? Can you explain why?

1. What are your expectations and biggest concerns?
2. How hopeful are you that your condition will get better?

Is there anything else that you would like to say that we may have not discussed or covered already?
