# Supplementary material for: “It’s very saddening, you keep on wondering when the symptoms will be over”: A qualitative study exploring the long-term chikungunya disease impact on daily life and well-being, 6 years after disease onset
Source: PLoS Negl Trop Dis. 2023 Dec 6;17(12):e0011793. doi: 10.1371/journal.pntd.0011793 (PMC10699624; doi:10.1371/journal.pntd.0011793)
Supplement: S1 Table — (DOCX) [file pntd.0011793.s002.docx]

**S1 Table. Code list: themes, codes, and illustrative quotes related to experiences of physical impact.**

| **Well-being category** | **Themes** | **Codes** | **Illustrative quotes** |
| --- | --- | --- | --- |
| **Experience of physical impact** | **Restricted physical functioning** | **Physical limitation and impairment upper body** | “You know what I have noticed?…You know, like your [talking in third person] hands let things go [let things fall]. Like it [hand] has no strength to open things.” (Pt. 12; Female 48-year-old, arthralgia and joint weakness in wrists and hands, and joint stiffness in knees) |
|  |  | **Physical limitation and impairment lower body** | “I used to walk a lot, now I can’t walk, how I am supposed to. I used to walk really long, but now I can maybe walk from here to there [pointing at the end of the street approximately 100 meters further].” (Pt. 3; Female 55-year-old, arthralgia in knees and joint swelling in knees and ankles) |
|  |  | **Pain after immobility** | “As soon as I stay bent down for too long or sit too long, my bones [joints] will hurt.” (Pt. 9; Female 44-year-old, arthralgia in the back and fingers, and joint weakness in wrists and hands) |
|  |  | **Stiffness after immobility during the day** | “I still feel the pain, my body [joints] is painful and stiff. That is why when I come home after work and if I have something to do, I don’t like to come home and sit down, because my body [joints] will get stiff. […]. They say whenever the blood is warm, you can do things [expression of increased endurance when moving].” (Pt. 13; Female 56-year-old, arthralgia and joint stiffness in the lower back and ankles) |
|  | **Limitations in activities of daily life** | **Morning stiffness** | “The pain is…you know when you are at rest it becomes a sickness of rheuma [rheumatoid arthritis]. When you have rheuma [rheumatoid arthritis] you have pain when you wake up and when you start to move it [pain] lessens….And every time I compare myself with…how do you call those animals? The reptiles [cold-blooded reptiles]…in the morning I don’t do much [due to stiffness] and during the day when the warmth is coming out [ambient temperature is rising] I will get a sort of speed.” (Pt. 7; Female 54-year-old, arthralgia in hands, fingers, hips, knees, and ankles) |
|  |  | **Interference daily functioning** | “I can’t lift up my arm, that is why I told my general practitioner…the day that I can eat at the dining table with my arm on the table, I will feel that I am [physically] good…but till this day I am eating with the plate here [on the lap and not on dinning table].” (Pt. 10; Female 77-year-old, arthralgia in shoulder, knee and toe, joint stiffness in knee, and joint swelling in ankle) |
|  |  | **Challenges with maintaining weight or weight gain** | “It [persistent symptom] hampers me to walk. I want to go and walk at the stadium near by as well, but I will not go, because when the pain hampers me, I can’t walk. My belly has become bigger [gain weight], but I can’t walk. […]. I can walk, but I can’t walk a lot [for long], cause when I walk a lot I need to sit down.” (Pt. 18; Male 58-year-old, myalgia in thigh and hamstring) |
|  |  | **Investment household appliances** | “No [can’t wring wet clothes]!...My son has bought an automatic washing machine [with spinner] for me. I throw the clothes in it [spinner] and put them [clothes] on the clothes line.” (Pt. 20; Female 62-year-old, arthralgia and joint cramps in wrist, and joint stiffness in hips, knees, and ankles) |
|  |  | **Interference daily activities** | “If I am doing household things, I need to sit down and swing my legs back and forth [for pain alleviation] to be able to continue with the things [domestic tasks]. If I clean the house the whole day… from front to back, I will have pain in my legs [ankles] the entire night.” (Pt. 1; Female 56-year-old, arthralgia in ankles) |
