# Supplementary material for: “It’s very saddening, you keep on wondering when the symptoms will be over”: A qualitative study exploring the long-term chikungunya disease impact on daily life and well-being, 6 years after disease onset
Source: PLoS Negl Trop Dis. 2023 Dec 6;17(12):e0011793. doi: 10.1371/journal.pntd.0011793 (PMC10699624; doi:10.1371/journal.pntd.0011793)
Supplement: S2 Table — (DOCX) [file pntd.0011793.s003.docx]

**S2 Table. Code list: themes, codes, and illustrative quotes related to experiences of psychological impact.**

| **Well-being category** | **Themes** | **Codes** | **Illustrative quotes** |
| --- | --- | --- | --- |
| **Experience of psychological impact** | **Altered emotional state** | **Mood change due to arthralgia** | “I noticed that I have become irritable, like stressed. […]. Some people have told me…you are very irritable, or some people tell me…you are annoying people [with by being stressed]…but how will you [talking in third person] tell them that you had that [chikungunya], maybe they will tell you that you are crazy…that you are blaming it [chikungunya].” (Pt. 12; Female 48-year-old, arthralgia and joint weakness in wrists and hands, and joint stiffness in knees) |
|  |  | **Psychological impact** | “You [talking in third person] aren’t happy, you aren’t living happy…With pain, with pain, with pain, you don’t live happilly…I for sure don’t.” (Pt. 20; Female 62-year-old, arthralgia and joint cramps in wrist, and joint stiffness in hips, knees, and ankles) |
|  |  | **Fear and frustration when symptoms re-occur** | “When the symptoms re-occur I will say…Will this thing [chikungunya disease] never go away? When will this thing get out of my system completely? […]. I knew that the symptoms can take long [persist], but I didn’t expect them to take this long! I have this [persistent symptoms] for more than 5 years now!” (Pt. 9; Female 44-year-old, arthralgia in the back and fingers, and joint weakness in wrists and hands) |
|  | **Fear of walking and running** | **Vigilance physical limitation** | “When my ankles are swollen its painful, very painful. I feel the pain in my ankles, and when I walk it will give me the sensation that my ankles and knees are unstable. I need to watch out very carefully how I step, because it seems that they [ankles and knee] loose strength.” (Pt. 3; Female 55-year-old, arthralgia in knees and joint swelling in knees and ankles) |
|  | **Psychosocial aspects footwear adaptations** | **Shoe adaptation** | “It bothers me [not being able to wear high heels], it really bothers me. […]. It’s like my knees can’t hold it, your [talking in third person] knees can’t hold it if you are on high heels.” (Pt. 11; Female 35-year-old, arthralgia in the wrists, hands, and knee) |
|  |  | **Emotional impact footwear** | “I love shoes with ankle straps, I find it very upsetting that I can’t wear shoes that wrap around the ankle anymore…because my legs may be normal now and later it [leg] will be swollen, so I won’t put a strap around it, because then it will be easier to see that it’s swollen.” (Pt. 17; Female 56-year-old, arthralgia in wrists, fingers, knees and ankles, joint locking of wrists, fingers and ankles, joint cramps in fingers, and joint swelling of ankles) |
|  | **Uncertainty about disease progression and future health** | **Future health perspective** | “I think that if it [persistent symptom] never ever goes away, I will start to say that I have rheumatoid arthritis…and maybe I don’t have it [rheumatoid arthritis], but I will start thinking that, but that is only if it [persistent symptom] never ever goes away…but if it [persistent symptom] goes away is something that I also don’t know.” (Pt. 1; Female 56-year-old, arthralgia in ankles) |
|  |  | **Health perspective ageing** | “I think I will still have pain in 2 years, because when you become older you don’t know how the pain will evolve.” (Pt. 18; Male 58-year-old, myalgia in thigh and hamstring) |
