# Supplementary material for: “It’s very saddening, you keep on wondering when the symptoms will be over”: A qualitative study exploring the long-term chikungunya disease impact on daily life and well-being, 6 years after disease onset
Source: PLoS Negl Trop Dis. 2023 Dec 6;17(12):e0011793. doi: 10.1371/journal.pntd.0011793 (PMC10699624; doi:10.1371/journal.pntd.0011793)
Supplement: S3 Table — (DOCX) [file pntd.0011793.s004.docx]

**S3 Table. Code list: themes, codes, and illustrative quotes related to experiences of social impact.**

| **Well-being category** | **Themes** | **Codes** | **Illustrative quotes** |
| --- | --- | --- | --- |
| **Experience of social impact** | **Social isolation and impaired relational maintenance** | **Symptoms interference social activities** | “A lot of times we [group of colleagues] were getting from work to go walking at [name of sports stadium]…I don’t participate with that anymore. […]. I have stopped because of my ankle.” (Pt. 19; Female 49-year-old, arthralgia and joint weakness in wrists and ankles) |
|  |  | **Social isolation** | “The day I wake up with pain…I will have pain and I won’t talk and people don’t understand, and I don’t think it’s worthwhile to talk. I will just remain silent for others not to tell me anything…and I find that I isolate myself quite often.” (Pt. 12; Female 48-year-old, arthralgia and joint weakness in wrists and hands, and joint stiffness in knees) |
|  |  | **Symptoms interference social relationships** | “If you are my friend and you will come to visit me, if I am in pain I will tell you don’t get angry, but I will go and rest a while. I will tell you that with millions of love [in a loving way], but it depends if you accept it. If you are my friend and you keep on visiting me, you may feel that [name participant] treats me like she doesn’t want me to come to her home, I won’t go anymore. I don’t know what people may think, but I am honest in telling you [visitor] that [she needs to rest], cause I know what I am feeling [in pain].” (Pt. 15; Female 48-year-old, arthralgia in elbow, wrist and knee, and joint weakness in wrist and knee) |
|  | **Social dependency** | **Social dependency** | “When I come home [from work] I can’t do anything, not even cook. Do you think that I will stand and cook? I will eat bread… my daughter brings a meal for me each night now, my life changed dramatically.” (Pt. 20; Female 62-year-old, arthralgia and joint cramps in wrist, and joint stiffness in hips, knees, and ankles) |
|  |  | **Support: instrumental** | “Jars and other things that I normally used to open, I now need to ask someone to open them for me.” (Pt. 9; Female 44-year-old, arthralgia in the back and fingers, and joint weakness in wrists and hands) |
|  | **Challenges of social support** | **Talking about chikungunya with others** | “You know a lot of time people don’t know, certain people know that I have constant pain, because I don’t make it a topic [don’t bring it up], I don’t make it a topic. Most people don’t even know, because sometimes you will tell them [about persistent symptoms] and they will tell you... Hacht [expression when being annoyed] you are talking stupidity, there she comes again with her pain [downplaying]…so I rather not [bring it up].” (Pt. 7; Female 54-year-old, arthralgia in hands, fingers, hips, knees, and ankles) |
|  |  | **Understanding of others** | “No, not everyone understands, only a person who had chikungunya will understand. An individual who did not have it [chikungunya] will tell you your exaggerating…They will tell you…something since 2014/2015?… but even I did not expect that after 6 years I still will have complaints [rheumatic symptoms] because of it [chikungunya].” (Pt. 19; Female 49-year-old, arthralgia and joint weakness in wrists and ankles) |
|  | **At-work productivity loss** | **Symptoms interference work tasks** | “At work we have bags with saline solution…you need to put the system [screw connector of the tube] in [the bag connector] and you need to just twist and tighten it [tube to bag]…that [twisting wrist] I am tired of it, I am really really tired of it… or I need to put the bag down to be able to do it. It is actually a thing that you are able to do [twist] at once, but because of my wrist I am not able to do it at once…I need to do it little by little, and that’s a weakness [doesn’t do tasks well].” (Pt. 17; Female 56-year-old, arthralgia in wrists, fingers, knees and ankles, joint locking of wrists, fingers and ankles, joint cramps in fingers, and joint swelling of ankles) |
|  |  | **Work tasks aggravate symptoms** | “Sometimes you need to lift heavy things [pans] at work and then everything [joints] will get stiff.” (Pt. 13; Female 56-year-old, arthralgia and joint stiffness in the lower back and ankles) |
|  |  | **Maintaining body position at work** | “I work, but sometimes I can stand 2 hours long without sitting, but sometimes I will stand 1,5 hours and I need to sit down [to rest]…but not every day, each day is different.” (Pt. 18; Male 58-year-old, myalgia in thigh and hamstring) |
|  |  | **Presenteeism and productivity loss** | “At work when I feel that my hand doesn’t go, I will say hold on, hold on, hold on, I can’t do this [work task], I will let another person [colleague] do it for me. […]. I will do the things [work tasks] that I need to do at work, but much slower. They [colleagues] can think what they want to think, but I am the one that feels [the pain].” (Pt. 7; Female 54-year-old, arthralgia in hands, fingers, hips, knees, and ankles) |
|  | **Giving up recreation and leisure activities** | **Work interference social activities** | “If I go to my sister after work and the moment I sit and relax in the chair I will be in trouble [joint pain will increase], I won’t have strength afterwards [after sitting down] and that’s when I get angry, because I need to drive home…and I feel like… he eh [expression when something is unfavourable or bad] I can’t do this [driving], how will I accomplish this? No no, I don’t like that, that’s why when I finish working I will go home immediately.” (Pt. 17; Female 56-year-old, arthralgia in wrists, fingers, knees and ankles, joint locking of wrists, fingers and ankles, joint cramps in fingers, and joint swelling of ankles) |
